# Associations of medical outcomes with substances involved in suicide attempt cases age 50 and older reported to U.S. Poison Centers, 2016–2023

**DOI:** 10.3389/fpubh.2025.1505040

**Published:** 2025-03-26

**Authors:** Namkee G. Choi, Bryan Y. Choi, C. Nathan Marti, S. David Baker

**Affiliations:** ^1^Steve Hicks School of Social Work, The University of Texas at Austin, Austin, TX, United States; ^2^Department of Emergency Medicine, Philadelphia College of Osteopathic Medicine and Bayhealth Medical Center, Dover, DE, United States; ^3^Central Texas Poison Center, Temple, TX, United States

**Keywords:** older adults, poisoning, suicide attempt, antidepressants, benzodiazepines, opioids, cardiovascular drugs

## Abstract

**Background and aims:**

After a slight decline in suicide rates during the COVID-19 pandemic, suicide rates and suicide attempts in the U.S. have been increasing again in 2022 and 2023. Compared to younger age groups, the 50+ age group has significantly higher rates of serious medical outcomes from suicide attempts. In this study, we examined the medical outcome severities associated with different classes of substances involved in suicide attempt cases age 50 and older who were reported to poison centers.

**Methods:**

We used the America's Poison Center's National Poison Data System from 2016 to 2023 (*N* = 335,171 cases age >50). Following descriptive statistics on the characteristics of suicide attempters and involved substances by medical outcomes (no/minimal effects, moderate effects, major effects, death, and unable to follow), we fitted multinomial and binary logistic regression models to examine the associations of medical outcomes with involved substances.

**Results:**

Of all cases, 22.5% used antidepressants, 21.1% benzodiazepines, 16.4% cardiovascular drugs, and 12.5% prescription opioids; 44.1% had no/minimal effect, 37.9% had a moderate effect, 12.5% had a major effect, 1.2% resulted in death, and 4.4% were unable to follow, but the proportions of major effects and death were higher in 2022–2023 than in 2016–2017. Tricyclic antidepressants were associated with the highest risks of major effects [relative risk ratios (RRR) = 5.57, 95% CI = 5.26–5.90] and death (RRR = 4.26, 95% CI = 3.67–4.94). Large RRRs were also shown for bupropion and serotonin-norepinephrine reuptake inhibitors for major outcomes and death. Cardiovascular drugs, opioids, and muscle relaxants were also associated with consistently higher risks of moderate and major effects and death. Our results also show that older ages were associated with higher death rates and that female sex was associated with higher odds of moderate/major effects compared to minimal effects but lower odds of major effects/death.

**Implications:**

Healthcare providers, including pharmacists, can play an important role in promoting medication safety for older adults. Healthcare workers need training in assessing and recognizing signs of suicide risk in older adults who are prescribed antidepressants and sedatives. Our findings also point to the importance of better non-pharmacological chronic pain management than reliance on opioids.

## Introduction

According to the U.S. Centers for Disease Control and Prevention (CDC), age-adjusted suicide rates increased by 37% between 2000 and 2018 to reach 14 per 100,000 population, decreased by 5% between 2018 and 2020 but returned to the 2018 level in 2022 when nearly 49,500 individuals died by suicide (1). The 85 and older group (23.0 per 100,000 in 2022) has the highest suicide rate, followed by the 75–84 age group (20.3 per 100,000) and the 50–60 age group (19.3 per 100,000), with much higher rates among males than females (1, 2).

The CDC and the Substance Abuse and Mental Health Services Administration data also show that 1.6 million adults (including 410,000 individuals age 50+) attempted suicide in 2022, an increase from 1.4 million (including 304,000 individuals age 50+) in 2017 or prior years (2006–2016) (1, 3, 4). The rate of attempted suicide was highest among those ages 18–25 (2.1%), followed by those ages 26–49 (0.5%) and those age 50 or older (0.3%) (3). However, compared to younger age groups, the 50+ age group has significantly higher rates of healthcare facility admissions and serious medical outcomes, including death, from suicide attempts (5, 6). A study also showed that between 2011 and 2020, the 65+ age group had the largest increase in emergency department visits for suicide attempts and intentional self-harm, with an average annual percent change of 30.2% (7). Although most people (i.e., over 90%) who attempt suicide do not go on to complete suicide, a history of suicide attempts is one of the strongest risk factors for suicide death (8). Thus, a better understanding of individuals who attempted suicide and risk factors is imperative for developing effective suicide prevention strategies.

A systematic review found that depression and other psychiatric disorders and poor physical health and disability are risk factors for both suicide attempts and completed suicide in late life globally; however, specific risk factors for suicide attempts include psychoactive medication use and drug poisoning, while male sex, stressors/bereavement, living alone, and lethal means (firearm and hanging) were more significant for predicting completed suicides (9). Other studies have also shown severe depressive symptom severity, anxiety, post-traumatic stress disorder, substance use disorders, chronic illness, frailty, disability, and bodily pain as significant correlates of suicidal behaviors in mid-life and older adults (10–19).

For older adults with depression, other psychiatric illnesses, and/or physical health problems, drug poisoning as a suicide attempt is facilitated by easy access to antidepressants, opioids, benzodiazepines, and other medications at home (20). According to the National Poison Data System (NPDS) that is compiled and managed by America's Poison Centers, the overall rate of calls received by poison centers for suspected suicides and nonfatal suicide attempts involving antidepressants increased from 17.4 per 100,000 U.S. population in 2000 to 28.4 in 2019, with selective serotonin reuptake inhibitors (SSRIs) being most commonly involved and tricyclic antidepressants (TCAs) being associated with the largest proportion of serious medical outcomes including death (5). The NPDS also showed that although the number of prescription opioid-involved suicide attempt cases age 50 and older decreased between 2015 and 2020 as U.S. opioid prescription rates declined, prescription opioids were associated with a higher likelihood of death and other serious medical outcomes (21). A majority of benzodiazepine poisoning calls to poison centers for those age 50 and older between 2015 and 2022 were suicide attempt cases (22). A previous study of over 421,000 drug-poisoning suicidal acts in 2011 and 2012, resulting in nearly 22,000 deaths, showed that 15.4%−17.3% involved opioids and 19.6%−22.5% involved benzodiazepines, but opioids were the most commonly identified agents in fatal suicide poisonings (33.3%−47.8%), followed by barbiturates, antidepressants, antidiabetics, calcium channel blockers, and alcohol (23).

Drug poisoning as a means for suicide attempts and self-harm often involves multiple prescription medications (24). Substantial proportions of suspected suicide attempt cases who were middle-aged and older adults used benzodiazepines and opioids concurrently (6, 25). Long-term (prescribed for ≥90/180 days) use of benzodiazepines and opioids and co-prescription of ≥3 psychoactive medications were also associated with death by suicide among women veterans age 50 and older (26). Analysis of statewide medical claims data found that the risk of suicide attempt was sharply elevated among patients with psychiatric conditions other than anxiety who were prescribed more frequent and higher opioid doses, indicating the synergic associations among pain, psychiatric problems, and opioids in suicide attempt (27).

Given the significant increase in poisoning suicide attempts among older adults in recent years, more research on the types of prescription medications and other substances used and associated medical outcomes is needed. In the present study, we used 2016–2023 NPDS cases age 50 and older to examine the classes of substances (antidepressants, antipsychotics, prescription opioids, benzodiazepines, cardiovascular drugs, other prescription and over-the-counter medications, and alcohol) frequently involved in suspected suicide attempts and the associations between medication classes and medical outcomes (no/minimal effect, moderate effect, major effect, and death).

## Materials and methods

### Data source

This is an observational study of 8 years (January 1, 2016 through December 31, 2023) of pooled NPDS data from 55 America's Poison Centers with locations in all 50 states, the District of Columbia, and Puerto Rico [see NPDS website (https://poisoncenters.org/national-poison-data-system) or Gummin et al. (28) for detailed descriptions]. We chose these years to reflect the fluctuations in the overall suicide rates, as described earlier. We focused on closed cases age 50 and older whose exposure reason was suspected suicide attempt, i.e., intentional poisoning for self-harm and suicidal intent. The NPDS lists all co-used substances in each case, allowing a comprehensive analysis of these substances. The NPDS lists cases, not individuals; however, the extent to which these cases include duplicate individuals is minimal, as poison center specialists are trained to detect duplication and correct it as soon as it is discovered. Of the 343,895 NPDS cases in which suicide attempt was reported to be the exposure reason, we excluded 271 confirmed non-exposure cases and 8,453 cases with unrelated effects (i.e., the exposure not responsible for the reported effect), resulting in 335,171 cases for this study. Since the majority (97% in this study) of these cases were reported to Poison Centers from healthcare facilities or Emergency Medical Services (EMS), suicide attempts were likely to have been validated. Based on the authors' institutional review board guidelines, analysis of these de-identified data was exempt from the authors' institutional review boards' review.

### Measures

#### Antidepressant medication

The NPDS includes 31 codes for prescription antidepressants. To examine the effects of different classes of antidepressants, we grouped them into serotonin antagonist and reuptake inhibitors (SARIs: trazodone and nefazodone), selective serotonin reuptake inhibitors (SSRIs), serotonin and norepinephrine reuptake inhibitors (SNRIs), tricyclic antidepressants (TCAs), norepinephrine and dopamine reuptake inhibitor (NDRI; bupropion), tetracyclic antidepressants (TeCAs), monoamine oxidase inhibitors (MAOIs), and other/unknown types.

#### Other substances

These included prescription opioids (23 codes); benzodiazepines (one code); drugs for cardiovascular diseases (33 codes, including angiotensin-converting enzyme inhibitors; angiotensin receptor blockers; antihypertensives; beta-blockers; calcium antagonists; alpha-blockers; antiarrhythmics; and cardiac glycosides); antipsychotics (three codes; atypical antipsychotics, phenothiazines, and loxapine); anticonvulsants (16 codes); muscle relaxants (eight codes); both prescription and over-the-counter antihistamines (12 codes); both prescription and over-the-counter nonsteroidal anti-inflammatory drugs (NASIDs; 10 codes); and alcohol. We included alcohol as it has been frequently co-used with other substances in older adults' suicide attempts (22).

#### Medical outcomes

The NPDS has the following medical outcome categories for human exposure: no effect; minor effect; moderate effect (signs or symptoms that were not life-threatening or had no residual disability or disfigurement); major effect (signs or symptoms that were life-threatening or resulted in significant residual disability or disfigurement); death; indirectly reported death (those that poison centers acquired information from medical examiners or media); not followed (since the outcomes were judged to be nontoxic or expected to have minimal clinical outcomes); and unable to follow (including cases that refused referral to a healthcare facility or left against medical advice) (28). The NPDS medical outcome is for each case (i.e., the exposed human being), not for each substance. In this study, we had the following five medical outcomes: no/minimal effects (including those who were not followed up for nontoxic exposure or expected minimal clinical outcomes); moderate effects; major effects; death (including indirectly reported death); and refusal of care. Indirectly reported cases tend to be sporadic, and 245 cases out of 333 cases in this study were from Arizona (which has included deaths from state vital statistics since 2017) (29). However, we decided to include these deaths to show completed suicide. We used major effects/death and serious medical outcomes/serious outcomes interchangeably in this study.

*Other covariates* were exposure year (grouped into 2016–2017, 2018–2019, 2020–2021, and 2022–2023 to examine potential changes in these two-year intervals), age group (50–59, 60–69, 70–79, 80–89, and 90+ years), gender, and the U.S. census region (based on each case's state of residence reported in the NPDS). We reported exposure location, call and management/care sites, and the number of all substances involved in each case for descriptive purposes only.

### Analysis

All analyses were conducted with Stata 18/MP (Stata Corp, College Station, TX). First, we examined changes over the 8 years in the number of suicide attempt cases. Second, we used descriptive statistics (Pearson's χ^2^ tests or ANOVA) to compare demographics and involved substances of all suicide attempt cases by medical outcomes. Third, we fitted a multinomial logistic regression model to examine the associations of medical outcomes (moderate effects, major effects, and death vs. no/minimal effect) with involved substances, controlling for demographic characteristics. Given unknown outcomes, we excluded cases that were unable to follow (i.e., lost to follow-up). Fourth, we fitted a binary logistic regression model to examine substances that were associated with major effects or death vs. moderate effects. As a preliminary diagnostic, variance inflation factor, using a cut-off of 2.50 (30), from linear regression models indicated no concerning multicollinearity among covariates. Multinominal and binary logistic regression results are reported as relative risk ratios (RRRs) and adjusted odds ratios (ORs), respectively, with 95% confidence intervals (CIs).

## Results

### Changes in the reported cases and demographic characteristics by medical outcomes

**Figure 1:**
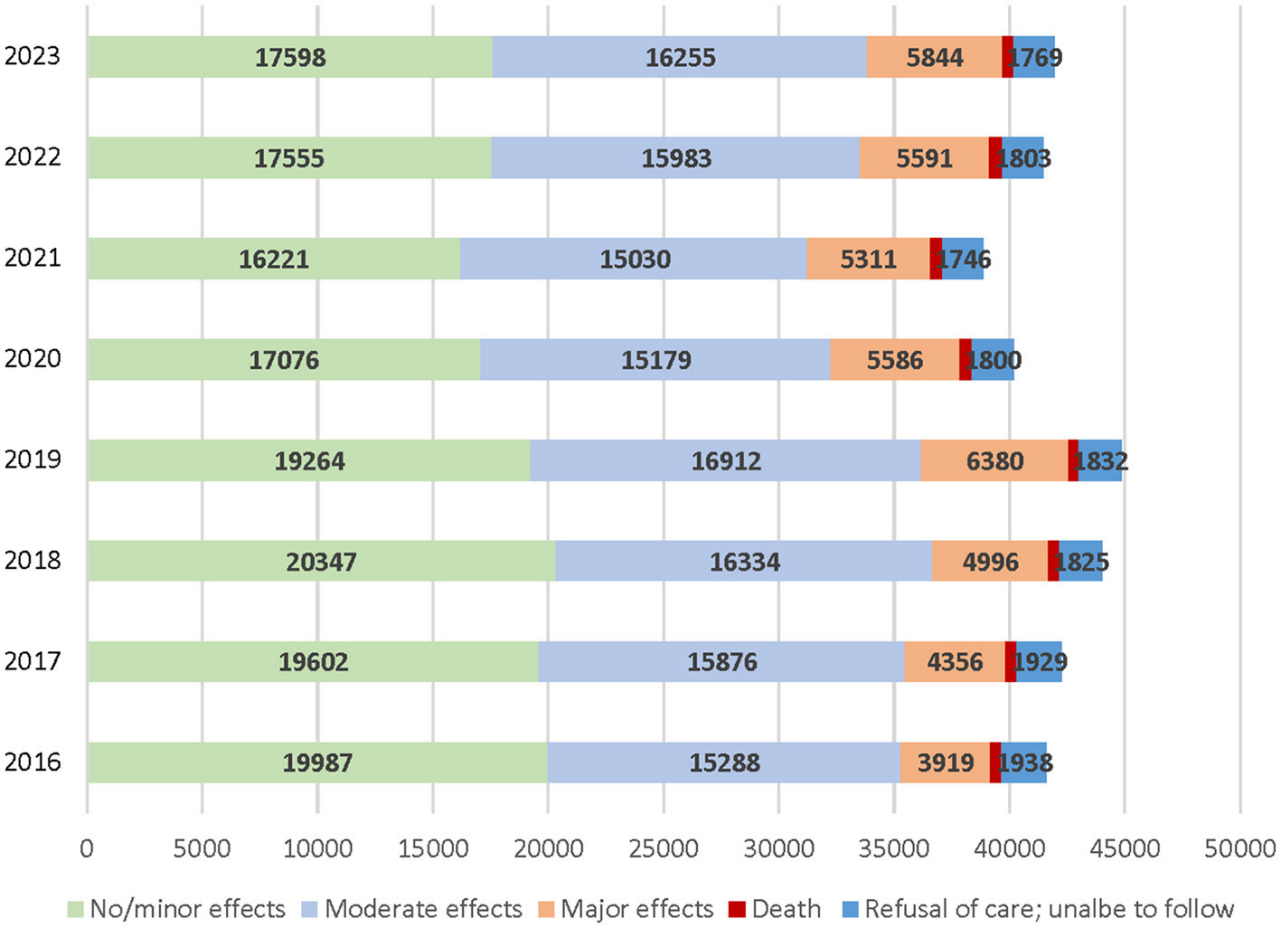
Number of suicide attempt cases by year and medical outcome.

Figure 1 shows that the number of suspected suicide attempt cases that were reported to poison centers decreased in 2020–2021 compared to 2018–2019 but increased in 2022–2023 relative to 2020–2021 but remained lower than in 2018–2019. Table 1 shows that of all cases, 44.1% had no/minimal effect, 37.9% had a moderate effect, 12.5% had a major effect, 1.2% resulted in death, and 4.4% were unable to follow, but the proportions of major effects and death were higher in 2022–2023 than in 2016–2017. Age distributions were 58.6% 50–59 years and 28% 60–69 years. Those age 80 and older were 3.7% of all cases but 10.7% of the deceased cases. A little over 60% of all cases were female, and 95% used poisoning at home. Of all cases, 94% were treated at a healthcare facility, but admissions to a critical care unit were higher for those with major effects (76.9%) and those who died (82.1%) compared to those with no/minimal effects (12.5%) and moderate effects (38.3%). More than three-quarters of the unable-to-follow cases refused referral to a healthcare facility or left against medical advice.

**Table 1:** Demographic characteristics of the suspected suicide attempt cases age 50 and older reported to poison centers, 2016–2023, by medical outcome.

	**All**	**No/minimal effects^a^**	**Moderate effects**	**Major effects**	**Death^b^**	**Unable to follow^c^**
	**335,171 (100%)**	**147,650 (44.05%)**	**126,857 (37.85%)**	**41,983 (12.53%)**	**4,039 (1.21%)**	**14,642 (4.37%)**
**Year (%)**						
2016–2017	25.02	26.81	24.57	19.71	23.50	26.41
2018–2019	26.51	26.83	26.21	27.10	24.02	24.98
2020–2021	23.57	22.55	23.81	25.96	26.32	24.22
2022–2023	24.90	23.81	25.41	27.24	26.17	24.40
**Age group (years, %)**						
50–59	58.63	59.84	59.01	55.00	39.42	58.81
60–69	28.04	27.25	28.23	30.24	31.49	26.99
70–79	9.60	9.19	9.33	11.03	18.37	9.69
80–89	3.10	3.07	2.91	3.14	8.79	3.43
90+	0.63	0.65	0.53	0.60	1.93	1.08
**Sex (%)**						
Female	60.85	60.27	61.43	61.93	57.81	59.32
Male	39.15	39.73	38.57	38.07	42.19	40.68
**Census region (%)**						
Northeast	15.40	14.59	16.31	16.48	17.42	11.88
Midwest	23.05	22.37	24.96	23.28	22.57	12.82
South	38.79	38.91	37.61	40.04	32.11	46.15
West	22.28	23.46	20.75	19.92	27.45	29.08
Puerto Rico/US Virgin Islands	0.48	0.67	0.37	0.27	0.45	0.06
**Exposure site (%)**						
Own home	94.66	95.08	94.86	93.47	93.24	92.49
Not own home	5.34	4.92	5.14	6.53	6.76	7.51
**Caller site (%)**						
Acute care hospital	90.50	91.89	93.26	93.97	87.15	43.65
Ambulance/rescue team	6.15	5.48	5.25	5.11	11.69	22.14
Own residence	2.82	2.21	1.22	0.69	0.77	29.54
Other	0.52	0.42	0.27	0.23	0.40	4.67
**Management site/Level of healthcare facility (HCF) care (%)**						
Managed on-site (not seen at HCF)	0.66	0.43	0.11	0.07	7.06	7.62
Treated/evaluated and released from HCF	19.48	29.16	15.16	4.89	5.42	5.09
Admitted to a psychiatric facility	24.51	37.70	18.95	5.17	0.05	1.95
Admitted to a noncritical care unit	19.18	17.46	25.91	12.00	4.83	2.74
Admitted to a critical care unit	30.86	12.47	38.28	76.88	82.05	5.87
Left AMA	4.48	2.43	1.53	0.97	0.59	61.88
Refused HCF referral	0.83	0.35	0.06	0.01	0	14.85

Omnibus Pearson's χ^2^ tests showed that all differences among the five medical outcomes were significant at p < 0.001.

^a^Including cases that were not followed because their outcomes were judged to be nontoxic or expected to have minimal clinical outcomes.

^b^Including both directly reported deaths (n = 3,706) and indirectly reported deaths (n = 333).

^c^Cases that refused referral, did not show up at healthcare facility, or left healthcare facility against medical advice.

### Substance exposure

Table 2 shows that 22.5% of all suicide attempt cases used antidepressants, and SARIs (almost all of them Trazodone) and SSRIs were the most common classes, followed by TCAs, SNRIs, and NDRI (bupropion). The next most involved medications were benzodiazepines (21.1% of all cases), cardiovascular drugs (16.4%), opioids (12.5%), antipsychotics (11.1%), anticonvulsants [11.1% (6.2% for Gabapentin)], muscle relaxants (7.0%), antihistamines (6.6%), and NSAIDs (3.8%). Alcohol was involved in 16.4% of all cases. Of all cases, 81.6% were exposed to at least one of these substances, and the average number of substances involved was 1.5 (SD = 0.72). The no/minimal effect group and the unable-to-follow cases were exposed to fewer substances than other outcomes groups.

**Table 2:** Substances involved in suspected suicide attempt by medical outcome.

	**All**	**No/minimal effects^a^**	**Moderate effects**	**Major effects**	**Death^b^**	**Unable to follow^c^**
	**335,171 (100%)**	**147,650 (44.05%)**	**126,857 (37.85%)**	**41,983 (12.53%)**	**4,039 (1.21%)**	**14,642 (4.37%)**
**Substances involved (%)**						
Antidepressants^d^	22.46	20.21	25.02	24.59	20.15	17.43
SARIs (mostly trazodone)	8.15	7.87	9.49	6.33	3.57	5.67
SSRIs	6.99	6.80	7.57	6.40	6.14	5.78
TCAs	2.98	1.55	3.36	6.96	5.08	1.97
SNRIs	2.93	2.61	3.23	3.33	3.17	2.23
NDRI (bupropion)	2.05	1.27	2.41	3.60	4.21	1.75
TeCAs	1.77	1.77	1.86	1.72	1.16	1.20
MAOIs	0.03	0.01	0.04	0.04	0.07	0.03
Other/unknown	0.07	0.07	0.07	0.05	0.10	0.07
Benzodiazepine	21.14	21.97	20.33	22.31	14.06	18.49
Alcohol	16.43	15.44	19.05	14.78	10.05	10.33
Cardiovascular drugs	12.50	10.32	14.27	14.04	31.86	9.36
Prescription opioids	12.17	10.11	12.68	17.90	17.93	10.46
Antipsychotics	11.12	9.28	12.85	13.91	6.44	7.92
Anticonvulsants	11.08	11.37	11.00	11.70	7.13	8.08
Muscle relaxants	7.00	4.78	8.29	11.63	5.27	5.31
Antihistamines	6.63	6.41	7.27	5.59	4.21	7.01
NSAIDs^e^	3.82	4.78	3.11	2.43	3.12	4.35
Involvement of any one of the above substances (%)	81.61	79.78	84.45	82.64	74.77	74.30
**Total No. of any of the above substances involved (*****n*** = **273,521)**						
Range	1–5	1–5	1–5	1–5	1–5	1.5
Mean (SD)	1.52 (0.72)	1.44 (0.66)	1.59 (0.74)	1.68 (0.81)	1.61 (0.78)	1.33 (0.60)
Median	1.0	1.0	1.0	1.0	1.0	1.0

Omnibus Pearson's χ^2^ tests showed that all differences among the five medical outcomes were significant at p < 0.001.

^a^Including cases that were not followed because their outcomes were judged to be nontoxic or expected to have minimal clinical outcomes.

^b^Including both directly reported deaths (n = 3,706) and indirectly reported deaths (n = 333).

^c^Cases that refused referral, did not show up at healthcare facility, or left healthcare facility against medical advice.

^d^SARIs: serotonin antagonist and reuptake inhibitors (99.9% of the cases involved trazodone; only 17 out of 28,289 cases in this category involved nefazodone); SSRIs, selective serotonin reuptake inhibitors; *TCAs*, tricyclic antidepressants; SNRIs, *serotonin and norepinephrine reuptake inhibitors;* NDRIs, norepinephrine and dopamine reuptake inhibitor; TeCAs, tetracyclic antidepressants; MAOIs, monoamine oxidase inhibitors; and other/unknown types of antidepressants.

^e^Non-steroidal anti-inflammatory drugs.

Additional analysis showed that antidepressant exposure did not substantially change between 2016 (21.9%) and 2023 (21.9%; *p* = 0.854), although it reached 23.2% in 2020 (during the COVID pandemic); however, steady declines were observed for benzodiazepine (25.4% in 2016 to 18.5% in 2023, *p* < 0.001) and opioid (15.1% in 2016 to 10.6% in 2023, *p* < 0.001) exposures during the study period. Compared to those who were not exposed to antidepressants, lower proportions of those who were exposed to antidepressants used benzodiazepines (22.5% of nonusers vs. 16.5% of users), opioids (14.0% vs. 5.9%), and cardiovascular drugs (12.9% vs. 11.3%; *p* < 0.001 for all comparisons). A slightly lower proportion of benzodiazepine users than non-users used opioids (11.7% vs. 12.3%, *p* < 0.001). Among opioid users, 20.4% co-used benzodiazepines and 10.9% co-used antidepressants. Alcohol use was higher among antidepressant users than nonusers (17.5% vs. 16.1%) and benzodiazepine users than nonusers (18.4% vs. 15.9%) but lower among opioid users than nonusers (11.2% vs. 17.2%; *p* < 0.001 for all comparisons).

### Substances associated with moderate and major effects and death vs. no/minimal effects

Table 3 shows that of the antidepressant classes, TCAs had the largest RRRs for moderate effects (RRR = 2.47, 95% CI = 2.34–2.60), major effects (RRR = 5.57, 95% CI = 5.26–5.90), and death (RRR = 4.26, 95% CI = 3.67–4.94). Large RRRs were shown also for bupropion [moderate effects (RRR = 1.98, 95% CI = 1.87–2.10), major effects (RRR = 3.26, 95% CI = 3.04–3.49), and death (RRR = 3.97, 95% CI = 3.37–4.67)], and SNRIs [moderate effects (RRR = 1.24, 95% CI = 1.19–1.30), major effects (RRR = 1.29, 95% CI = 1.21–1.37), and death (RRR = 1.27, 95% CI = 1.06–1.52)]. Trazodone, SSRIs, and TeCAs were associated with a higher risk of moderate effects compared to no/minimal effects, but they were either not associated with or associated with a lower risk of major effects and death.

**Table 3:** Associations of medical outcomes with substances involved: multinomial logistic regression results.

	**Moderate effects RRR (95% CI)**	**Major effects RRR (95% CI)**	**Death RRR (95% CI)**
	**Vs. no/minimal effect**		
**Year: vs. 2016–2017**			
2018–2019	1.07 (1.05–1.09)^***^	1.41 (1.36–1.45)^***^	1.00 (0.91–1.09)
2020–2021	1.15 (1.13–1.18)^***^	1.62 (1.57–1.68)^***^	1.26 (1.15–1.38)^***^
2022–2023	1.16 (1.14–1.19)^***^	1.62 (1.57–1.68)^***^	1.17 (1.07–1.28)^**^
**Age group: vs. 50–59 years**			
60–69	1.07 (1.05–1.09)^***^	1.20 (1.17–1.23)^***^	1.66 (1.54–1.79)^***^
70–79	1.11 (1.08–1.14)^***^	1.35 (1.30–1.40)^***^	2.72 (2.48–2.98)^***^
80–89	1.08 (1.03–1.13)^**^	1.22 (1.14–1.30)^***^	3.66 (3.24–4.14)^***^
90+	0.97 (0.88–1.07)	1.16 (1.00–1.33)^**^	3.84 (3.02–4.88)^***^
Female vs. Male	1.06 (1.04–1.07)^***^	1.04 (1.02–1.06)^**^	0.97 (0.91–1.03)
**Census region: vs. Northeast**			
Midwest	0.97 (0.95–0.99)^*^	0.90 (0.87–0.93)^***^	0.89 (0.81–0.99)^*^
South	0.85 (0.83–0.87)^***^	0.88 (0.85–0.91)^***^	0.71 (0.64–0.77)^***^
West	0.78 (0.76–0.80)^***^	0.73 (0.70–0.75)^***^	0.97 (0.88–1.07)
Puerto Rico/US Virgin Islands	0.55 (0.49–0.62)^***^	0.39 (0.32–0.48)^***^	0.60 (0.37–0.96)^*^
**Substances involved**			
**Antidepressants** ^**a**^			
Trazodone (SARI)	1.30 (1.27–1.34)^***^	0.91 (0.87–0.95)^***^	0.55 (0.47–0.65)^***^
SSRIs	1.13 (1.09–1.16)^***^	0.98 (0.93–1.02)	0.91 (0.79–1.03)
TCAs	2.47 (2.34–2.60)^***^	5.57 (5.26–5.90)^***^	4.26 (3.67–4.94)^***^
SNRIs	1.24 (1.19–1.30)^***^	1.29 (1.21–1.37)^***^	1.27 (1.06–1.52)^*^
Bupropion (NDRI)	1.98 (1.87–2.10)^***^	3.26 (3.04–3.49)^***^	3.97 (3.37–4.67)^***^
TeCAs	1.07 (1.01–1.14)^*^	1.02 (0.94–1.11)	0.67 (0.50–0.90)^**^
Other	1.57 (1.23–2.01)^***^	1.39 (0.96–2.00)	2.31 (1.07–5.01)^*^
**Benzodiazepines**	0.98 (0.97–1.00)	1.15 (1.12–1.18)^***^	0.70 (0.64–0.76)^***^
**Cardiovascular drugs**	1.61 (1.57–1.65)^***^	1.68 (1.62–1.74)^***^	4.07 (3.79–4.37)^***^
**Prescription opioids**	1.51 (1.47–1.55)^***^	2.28 (2.21–2.35)^***^	2.14 (1.96–2.33)^***^
**Antipsychotics**	1.61 (1.57–1.65)^***^	1.94 (1.87–2.01)^***^	0.91 (0.80–1.04)
**Anticonvulsants**	0.99 (0.97–1.02)	1.08 (1.04–1.12)^***^	0.71 (0.62–0.80)^***^
**Muscle relaxants**	2.00 (1.93–2.07)^***^	2.98 (2.86–3.10)^***^	1.42 (1.23–1.64)^***^
**Antihistamines**	1.30 (1.26–1.34)^***^	1.08 (1.04–1.12)^***^	0.86 (0.74–1.00)
**NSAIDs** ^**b**^	0.71 (0.68–0.73)^***^	0.59 (0.55–0.63)^***^	0.73 (0.61–0.88)^**^
**Alcohol**	1.36 (1.33–1.39)^***^	1.08 (1.05–1.11)^***^	0.80 (0.72–0.89)^***^
Model statistics	*N* = 320,162; Likelihood ratio χ^2^ = 20,004.31, df = 84; *p* < 0.001		

^a^SARIs: serotonin antagonist and reuptake inhibitors (99.9% of the cases involved trazodone; only 17 out of 28,289 cases in this category involved nefazodone); SSRIs, selective serotonin reuptake inhibitors; *TCAs*, tricyclic antidepressants; SNRIs, *serotonin and norepinephrine reuptake inhibitors;* NDRIs, norepinephrine and dopamine reuptake inhibitor; TeCAs, tetracyclic antidepressants; MAOIs, monoamine oxidase inhibitors (MAOIs); and other/unknown types of antidepressants.

^b^Non-steroidal anti-inflammatory drugs.

^*^p < 0.05.

^**^p < 0.01.

^***^p < 0.001.

Table 3 also shows that cardiovascular drugs, opioids, and muscle relaxants were associated with consistently higher risks of moderate and major effects and death. Antipsychotics, antihistamines, and alcohol were also associated with a higher risk of moderate and major effects but not death. Benzodiazepines and anticonvulsants were associated with a higher risk of major effects but a lower risk of death. NSAIDs were associated with a lower risk of moderate and major effects and death.

Of the covariates, compared to 2016–2017, subsequent years were associated with higher risks of moderate and major effects and death. Compared to the 50–59 age group, older groups had higher risks of moderate and major effects and progressively higher risks of death. Female sex was associated with a higher risk of moderate and major effects but not death. The results also show that the Northeast region was associated with higher risks of moderate and major effects and death than the other U.S. census regions.

### Substances associated with major effects or death vs. moderate effects

Table 4 shows that TCAs (OR = 2.22, 95% CI = 2.12–2.33), bupropion (OR = 1.68, 95% CI = 1.58–1.79), benzodiazepines (OR = 1.11, 95% CI = 1.08–1.14), cardiovascular drugs (OR = 1.17, 95% CI = 1.14–2.21), opioids (OR = 1.50, 95% CI = 1.46–1.55), antipsychotics (OR = 1.15, 95% CI = 1.11–1.19), anticonvulsants (OR = 1.06, 95% CI = 1.02–1.09), and muscle relaxants (OR = 1.43, 95% CI = 1.38–1.48) were associated with higher odds of major effects/death compared to moderate effects. On the other hand, trazodone, SSRIs, antihistamines, NSAIDs, and alcohol were associated with lower odds of major effects/death compared to moderate effects.

**Table 4:** Correlates of major effects or death compared to moderate effect: logistic regression results.

	**Major effects or death vs. moderate effects OR (95% CI)**
**Year: vs. 2016–2017**	
2018–2019	1.28 (1.24–1.32)^***^
2020–2021	1.37 (1.33–1.42)^***^
2022–2023	1.36 (1.31–1.40)^***^
**Age group: vs. 50–59 years**	
60–69	1.14 (1.11–1.17)^***^
70–79	1.31 (1.26–1.36)^***^
80–89	1.31 (1.23–1.39)^***^
90+	1.41 (1.24–1.62)^***^
Female vs. male	0.97 (0.95–0.99)^*^
**Census region: vs. Northeast**	
Midwest	0.93 (0.89–0.96)^***^
South	1.01 (0.98–1.05)
West	0.96 (0.93–0.99)^*^
Puerto Rico/US Virgin Islands	0.78 (0.64–0.95)^*^
**Substances involved**	
**Antidepressants** ^**a**^	
Trazodone (SARI)	0.67 (0.65–0.70)^***^
SSRIs	0.86 (0.82–0.90)^***^
TCAs	2.22 (2.12–2.33)^***^
SNRIs	1.03 (0.97–1.09)
Bupropion	1.68 (1.58–1.79)^***^
TeCAs	0.96 (0.88–1.04)
Other	0.93 (0.67–1.29)
**Benzodiazepines**	1.11 (1.08–1.14)^***^
**Cardiovascular drugs**	1.17 (1.14–1.21)^***^
**Prescription opioids**	1.50 (1.46–1.55)^***^
**Antipsychotics**	1.15 (1.11–1.19)^***^
**Anticonvulsants**	1.06 (1.02–1.09)^**^
**Muscle relaxants**	1.43 (1.38–1.48)^***^
**Antihistamines**	0.82 (0.78–0.86)^***^
**NSAIDs**	0.85 (0.79–0.90)^***^
**Alcohol**	0.78 (0.76–0.81)^***^
Model statistics	*N* = 172,702; Likelihood ratio χ^2^ = 4,281.35, df = 28; *p* < 0.001

^a^SARIs, serotonin antagonist and reuptake inhibitors (99.9% of the cases involved trazodone; only 17 out of 28,289 cases in this category involved nefazodone); SSRIs, selective serotonin reuptake inhibitors; *TCAs*, tricyclic antidepressants; SNRIs, *serotonin and norepinephrine reuptake inhibitors;* NDRIs, norepinephrine and dopamine reuptake inhibitor; TeCAs, tetracyclic antidepressants; MAOIs, monoamine oxidase inhibitors (MAOIs); and other/unknown types of antidepressants.

^b^Non-steroidal anti-inflammatory drugs.

^*^p < 0.05.

^**^p < 0.01.

^***^p < 0.001.

Of the covariates, compared to 2016–2017, subsequent years were associated with higher odds of major effects/death. Older ages, compared to 50–59 years, were associated with greater odds, but females had lower odds of major effects/death. Compared to the Northeast region, the other regions, except the South region, were associated with lower odds of major effects/death.

## Discussion

Given the high suicide rates among older adults, this study focused on cases age 50 and older who were reported to poison centers between 2016 and 2023 for suspected suicide attempts and examined the associations between medical outcome severities and the substances used in the attempts. The findings show that antidepressants and benzodiazepines were most commonly involved (i.e., 23% and 21%) in poison-center-reported suicide attempt cases age 50 and older between 2016 and 2023. Opioids, cardiovascular drugs, antipsychotics, and anticonvulsants were also found in more than one out of 10 cases, and muscle relaxants, antihistamines, and NSAIDs were found in 4%−7% of cases. Psychoactive medication use was expected, as depression, anxiety, other psychiatric disorders, and chronic pain are risk factors for suicidal behaviors (9). Our findings also show that cardiovascular conditions were common among these suicide attempters.

Our findings related to the serious medical outcomes of TCAs are consistent with those of previous research discussed earlier. Additionally, we found SNRIs and bupropion were associated with a higher likelihood of more serious effects (moderate and major effects and death). Trazodone, SSRIs, and TeCAs were associated with the likelihood of moderate effects but not with major effects or death. TCAs' cardiovascular effects including blood pressure fluctuation as well as orthostatic hypotension, conduction delay, and a potent arrhythmic effect, especially among those with preexisting cardiovascular conditions, have been well established (31, 32). In our study, 12.5% of all cases but 31.9% of the deceased cases involved cardiovascular drugs, suggesting that those with pre-existing cardiovascular conditions had higher odds of dying from their poisoning suicide attempts.

SNRIs, particularly venlafaxine, also carry a greater risk of hypertension, possibly related to greater effects on the sympathetic nervous system (33). Bupropion was found to have a favorable cardiovascular profile compared to TCAs (34). However, with its expanding indications including smoking cessation, weight loss, attention-deficit/hyperactivity disorder, seasonal affective disorder, and amphetamine dependence, bupropion is subject to intentional misuse, and excessive doses may cause altered mental status, seizures, and dysrhythmias (35, 36). In the 2019–2020 NPDS, a small proportion of bupropion exposure cases (57% of them being suicide attempt cases) experienced an adverse cardiovascular event (vasopressor use, ventricular dysrhythmia, myocardial injury, or cardiac arrest), with increasing age as a significant factor (37).

As aforementioned, benzodiazepines are often the most frequently involved drugs in suicide attempts (38, 39). While the lethality of benzodiazepines may be lower than opioids (23), benzodiazepine users were found to have higher rates of healthcare utilization and were more likely to attempt and complete suicide than non-users (38, 40). Although the proportions exposed to benzodiazepines decreased over the study period, the rate of benzodiazepine exposure was still high in 2023 and a likely reflection of the continued high rates of benzodiazepine prescribing practice despite being contraindicated for older adults. The American Geriatric Society's Beers Criteria for Potentially Inappropriate Medication Use in Older Adults recommends that benzodiazepines and non-benzodiazepine hypnotics (e.g., zolpidem) should be avoided in all older adults due to the risk of cognitive impairment, sedation, delirium, falls, fractures, motor vehicle crashes, and dependence (41, 42). Although long-term benzodiazepine use is especially contraindicated for older adults, an earlier study based on large longitudinal prescription data found that about a third of benzodiazepine prescriptions for those age 65 years and older were for longer than 120 days per year (43). Research has also shown that adults aged 50 and older were more likely to misuse benzodiazepines (defined as using more often than prescribed) than younger adults (44).

Our findings also show that benzodiazepine exposure was associated with a higher likelihood of moderate vs. no/minimal effects and of major effects/death vs. moderate effects. In addition to cognitive and functional impairment, falls, and dependence, the adverse effects of both therapeutic-dose and long-term use of benzodiazepines in terms of aspiration and pneumonia, potentially lethal respiratory depression, and increased frailty, especially when co-used with opioids, depression and other psychiatric disorders, alcohol and other substance use disorders, and mortality have been well-established (45–49).

Consistent with previous research findings, our findings show that prescription opioids are associated with a high likelihood of serious medical outcomes in suicide attempt cases age 50 and older. As discussed, chronic pain is associated with an increased risk of suicide. Even though opioids are often prescribed to treat chronic pain, pain may predispose people to riskier use of opioids and opioid use disorders, which in turn increase the risk of suicide (50, 51). Higher doses of opioids have been linked to higher suicide risks (52). Lethal overdose with opioids occurs through excessive activation of the mu-opioid receptors in the locus coeruleus neurons in the brain, resulting in central nervous system (CNS) depression, somnolence, hypotension, and potentially lethal respiratory depression (53, 54). The presence of co-intoxicants with a CNS depressant (mainly benzodiazepines and alcohol) is also a significant confounder (55), although an analysis of large commercial claims and encounters databases from 2014 to 2016 did not find evidence of increased risk of suicide attempts due to interaction between opioids and benzodiazepines (26). Research has also shown it is difficult to discern whether an overdose was a suicide attempt or unintentional (56); however, opioid exposure, especially in high doses and concomitantly with other substances, would still have contributed to serious medical outcomes among older adults with suicidal intentions especially when comorbid psychiatric conditions were not addressed (57). As described, one out of five opioid users in this study also used benzodiazepines, and one out of 10 co-used antidepressants.

With respect to other medications, our findings are also similar to those of another study that examined poisoning severity associated with a range of medications in suicide attempt patients, ages 13–65, who were treated at a large academic hospital (58). The study showed that quetiapine (antipsychotic) and diphenhydramine (antihistamine) were associated with moderate/severe poisoning severity, but anticonvulsants were not. A review of research on the associations between antipsychotics and suicidal behaviors shows that antipsychotics, in general, tend to decrease suicidal behaviors for people with schizophrenia or schizoaffective disorder but increase such behaviors for people with bipolar disorders; however, the effects also varied between first- and second-generation antipsychotics and among specific medications and specific study participant pools (59). Thus, caution is needed in interpreting our findings related to antipsychotics. Anticonvulsants, especially gabapentin, have been used for chronic neuropathic pain, fibromyalgia, and migraine headache; however, they were not found to increase the risk for suicide attempt (60, 61), although adverse cardiovascular effects, dizziness, and somnolence (62, 63) have been shown. The significant association between anticonvulsants and serious medical outcomes in our study may stem from the conditions that the anticonvulsants were used to treat.

Our findings show that alcohol was associated with higher risks of moderate and major effects compared to no/minimal effects but a lower risk of death and that alcohol was associated with a lower risk of major effects/death compared to moderate effects. Since older adults are vulnerable to potentially serious alcohol-medication interactions, particularly those involving cardiovascular and CNS agents (64), we expected that alcohol would exacerbate and contribute to serious medical outcomes. However, our findings did not show that. A previous NPDS-based study of older suicide attempt cases involving benzodiazepines found that alcohol was not associated with major effects/death controlling for other substances (22). Pre-existing alcohol use disorder or heavy drinking behavior could worsen outcomes, but more research is needed to examine alcohol's effect on poisoning suicide attempts.

Our findings show that the likelihood of serious outcomes increased over the years compared to 2016–2017. Even though the number of suicide attempts decreased during the COVID-19 pandemic (2020–2021), the likelihood of serious outcomes still increased. It is unclear if the increase was due to increasing poisoning severity or other factors. The higher likelihood of severe outcomes, especially death, among those in their 80s and 90s was not surprising. Along with their likely higher levels of multi-morbidity and associated frailty and chronicity of medication intake, these age groups of older adults, especially males, have higher suicide completion rates as they generally have greater intent to die (65). Our findings show that females than males tend to have higher odds of moderate/major effects than minimal effects, but they have lower odds of major effects/death than moderate effects. The reasons for regional differences (i.e., higher odds of moderate/major effects and death in the Northeast) are unclear. A study based on the 2014–2019 National Vital Statistics System showed that mortality from drug poisoning was higher, but the suicide rate was lower in the Northeast than in the other regions (66). Research is needed to examine geographic differences in the rates of suicide attempts by poisoning.

There are some limitations related to the NPDS. First, since the NPDS includes only voluntarily reported cases, mostly by healthcare facilities that treated such cases, the generalizability of these findings is limited. Second, deaths among cases are likely underestimates as not all cases were followed up, and it is not clear if all reported deaths were related to substance use or from other causes. A recent study that analyzed the 2000–2020 suicide poisoning mortality in NPDS found that the NPDS cases averaged 11% (range 8%−16%) of those in the CDC's WONDER (67). Third, data that are telephone-reported to poison centers without medical record validation and toxicological confirmation may compromise validity. Fourth, a large proportion of cases had missing data on drug quantity and exposure chronicity, preventing analysis of these variables. Fifth, the lack of data on characteristics such as race, pre-existing and new health conditions, and substance use history precluded more detailed analyses. Studies have shown that new diagnoses of mild cognitive impairment and dementia increased suicide attempts and suicide in older adults (68, 69). We also want to acknowledge that medication class-based examinations rather than individual agents may be problematic as each medication differs. Future research needs to focus on the medical outcomes of individual medications.

This study demonstrates a stark public health crisis with many individuals age 50 and older utilizing self-poisoning in suicide attempts. The number of suicide attempt cases reported to poison centers is alarming, even with significant under-representation of the actual numbers. Even an attempt that did not lead to death can still incur serious emotional and physical effects on the attempters and can be a devastating event for their support system. Late-life suicide attempts and suicide must be prevented.

Despite its limitations, the study has the following implications for the prevention of suicide attempts. First, given the nature of drug poisoning largely involving prescription medications, healthcare providers can play an important role in promoting medication safety for older adults, including reducing easy access to potentially dangerous medications. Physicians need to regularly review and reduce unnecessary medications in older adults, especially those linked to high overdose risk. Pharmacists can play a critical role in counseling older adults on the safe use of medications and monitoring for signs of drug misuse or overprescription. Our study specifically shows high risks associated with TCAs, NDRI, benzodiazepines, opioids, and cardiovascular drugs. Patients prescribed these medications need ongoing monitoring for safety. Prescription drug monitoring programs can help track and regulate the use of medications that are frequently involved in suicide attempts. The uptake and effectiveness of the monitoring programs can be improved by addressing system-related barriers and negative end-user perceptions (70).

Second, the high prevalence of antidepressants and sedative use among suicide attempters calls for routine suicide risk screenings for those who were prescribed these medications. Healthcare providers require training to assess and identify suicide risk in this population accurately. Research has shown that primary care is an ideal setting to identify suicide risk and initiate psychosocial interventions for suicide prevention, with strong evidence for improved accessibility and effectiveness of such integrated approaches (71, 72). Despite the high prevalence of suicide attempts and suicide in the 50+ age group, older adults, in general, do not access the existing systems of behavioral health services due to stigma and the lack of availability and accessibility of such services (73–75). Telehealth can also improve access, particularly for those who are homebound or in rural areas (76). This underscores the need for insurance coverage for telepsychiatry services and reimbursement parity between in-person and telehealth mental health services. These and other suicide prevention policies are needed in a rapidly aging society.

Third, our study also points to the importance of alternative chronic pain management to reliance on opioids. Psychotherapy, such as cognitive behavioral therapy, is a promising option for older adults with chronic pain, as it also addresses the emotional impact of pain, which can influence pain perception and overall wellbeing. However, cognitive behavioral therapy, acceptance and commitment therapy, and mindfulness-based interventions produce only modest and time-limited benefits for older adults (77–80). Research is needed to find more effective psychotherapeutic interventions that can reduce pain intensity, improve function, and decrease the need for opioids in older adults. Physical and occupational therapy, exercise/movement therapy including Tai Chi and yoga, biofeedback, and alternative medicine such as acupuncture tailored to individual conditions also need to be more accessible. Prevention of opioid-related suicide and unintentional overdose also calls for better management of psychiatric comorbidities and more accessible treatment for opioid use and other substance use disorders, along with improving opioid prescription practices for pain management.

Fourth, in an ideal scenario, all suicide attempt cases need to be followed up after their healthcare service visits (which is akin to a crisis intervention) and provided ongoing supportive services so that suicide attempts would not be repeated. The NPDS does not include data on follow-up measures other than medical outcomes for the suicide attempt cases. Of course, as indicated by the “unable-to-follow-up” cases, it is difficult if those individuals refuse any service. At a minimum, all those at risk of suicide attempt or re-attempt must be encouraged to call 988 or other hotlines in times of distress.

Suicide is a complex and multifaceted behavior with numerous points for intervention (81–83). Although our study lacked data on social support and other precipitants of suicide attempts, previous research and suicide prevention recommendations emphasized social and community support, as loneliness and isolation are major risk factors (84). Helping people with depression and other psychiatric conditions, frailty, and disability stay connected with the sources of social support via telephone support calls, technology-based programs, and other community-based social connectedness programs would be a helpful strategy.

## Conclusions

This study shows that the number of poisoning suicide attempt cases age 50 and older that were reported to poison centers increased again in 2022 and 2023 after a slight decline during the COVID pandemic in 2020–2021. The risk of serious medical outcomes was significantly higher in later years than in 2016–2017. More than one out of five suicide attempters between 2016 and 2023 were exposed to antidepressants, of which TCAs and bupropion were associated with higher risks of serious medical outcomes. One out of 10 cases were exposed to cardiovascular drugs, opioids, antipsychotics, and anticonvulsants, all of which were associated with significantly a higher likelihood of serious medical outcomes. Older ages, male gender, and residence in the Northeast region were also associated with higher risk of serious outcomes. These findings indicate the urgent need for more effective strategies to prevent suicide attempts and suicide for the 50+ age group, whose suicide rates keep increasing.

## Data Availability

The data analyzed in this study is subject to the following licenses/restrictions. The America's Poison Centers releases the National Poison Data System (NPDS) to investigators following a review of data request. The authors do not have permission to make the NPDS data set used in this study available to other investigators. Requests to access these datasets should be directed at: https://poisoncenters.org/national-poison-data-system.
